# STCRpy: a software suite for T-cell receptor structure parsing, interaction profiling, and machine learning dataset preparation

**DOI:** 10.1093/bioinformatics/btaf566

**Published:** 2025-10-10

**Authors:** Nele P Quast, Charlotte M Deane, Matthew I J Raybould

**Affiliations:** Oxford Protein Informatics Group, Department of Statistics, University of Oxford, Oxford OX1 3LB, United Kingdom; Oxford Protein Informatics Group, Department of Statistics, University of Oxford, Oxford OX1 3LB, United Kingdom; Oxford Protein Informatics Group, Department of Statistics, University of Oxford, Oxford OX1 3LB, United Kingdom

## Abstract

**Summary:**

Computational methods to guide early-stage TCR drug discovery and TCR repertoire informatics currently under-utilize solved and predicted structure data. Here, we streamline use of these data through an open-source python package for high-throughput TCR structure handling and analysis (STCRpy), facilitating analyses such as TCR:peptide-MHC complex orientation calculation/scoring, root-mean-square-distance evaluation, interaction profiling, and machine learning dataset curation.

**Availability and implementation:**

Freely available as a Python package at https://github.com/oxpig/STCRpy.

## 1 Introduction

T cell receptors (TCRs) direct the adaptive immune response by interacting with antigens, such as short peptide fragments, presented in mammalian cells by the Major Histocompatibility Complex (pMHC) (van der Merwe and Dushek 2011, Birnbaum *et al.* 2014, Murphy and Casey 2017). They are gaining increasing attention, as a basis for the antigen targeting arm of biotherapeutics, especially for cancer, whether in soluble or cellular modalities (Dolgin 2022, Shafer *et al.* 2022).

This coincides with the increased adoption of computational tools to streamline or reimagine the process of biotherapeutic molecule selection (Riley *et al.* 2016, Notin *et al.* 2024). In the TCR field, this centers around unsupervised clustering or supervised prediction models that operate on the amino acid sequence and aim to assign the specificity of an expanded clone from a natural T-cell receptor repertoire (Hudson *et al.* 2023).

These methods have so far shown promise in the context of thoroughly studied antigens, but are unable to generalize to unseen antigen contexts or more complex determinants of binding (Meysman *et al.* 2022, Hudson *et al.* 2023).

A strategy to improve the generalizability of models is to consider 3D structural information in addition to the sequence (Robinson *et al.* 2021). While a few structure-aware methods exist (Bradley 2023, Ghoreyshi and George 2023, Wang *et al.* 2024), the overheads involved in accessing structural information and processing TCR:pMHC pose predictions are a roadblock to new entrants to the field. While numerous immunoglobulin-specific software packages exist for sequence-based analysis of TCRs (e.g. Ye *et al.* 2013, Dunbar and Deane 2016), no such suite exists for annotating and processing TCR structure data, which are ever more abundant (Leem *et al.* 2018, Quast *et al.* 2025).

One bespoke processing step is calculating the relative orientation of the TCR to the pMHC, a property linked to the ability of cell-surface TCRs to trigger downstream signaling (Zareie *et al.* 2021) and to cross-reactivity and even auto-reactivity (Beringer *et al.* 2015, Gras *et al.* 2016). Many general protein-protein complex prediction software packages generate poses of signaling TCRs that fall outside the “canonical” angles consistent with this tenet of T cell biology. While methods have been proposed for calculating TCR geometries (Rudolph *et al.* 2006, Singh *et al.* 2020), there is no standard method with an accessible python interface, limiting reproducibility.

Here, we present STCRpy, a python package to automate the processing and characterization of solved and predicted TCR:pMHC complexes or *apo* TCRs.

## 2 Implementation

STCRpy is pip-installable on Linux and Mac OSX operating systems, has been unit tested to assess the functionality of all modules (described below) and is provided with a python API and as a command line interface with full documentation.

### 2.1 Parsing, annotation, and interaction analysis

TCR:pMHC complexes can be parsed from local PDB or MMCIF files, or pulled directly from STCRDab (Leem *et al.* 2018) or the PDB (Berman *et al.* 2000). Protein chains are first annotated using a modified version of ANARCI (Dunbar and Deane 2016) able to classify MHC and MHC-like sequences as well as immune receptors (available as https://github.com/oxpig/anarci-mhc). All TCRs are numbered and annotated by region according to the IMGT system/definitions (Lefranc *et al.* 2005). TCRs and antigens are then paired by distance into separate “TCR” objects, accounting for multiple TCR:pMHC copies per PDB file. As an extension of the BioPython PDB module (Cock *et al.* 2009), STCRpy parses structures in a hierarchical manner (from “model” to “chain” to “residue” to “atom”).

**Figure 1.**
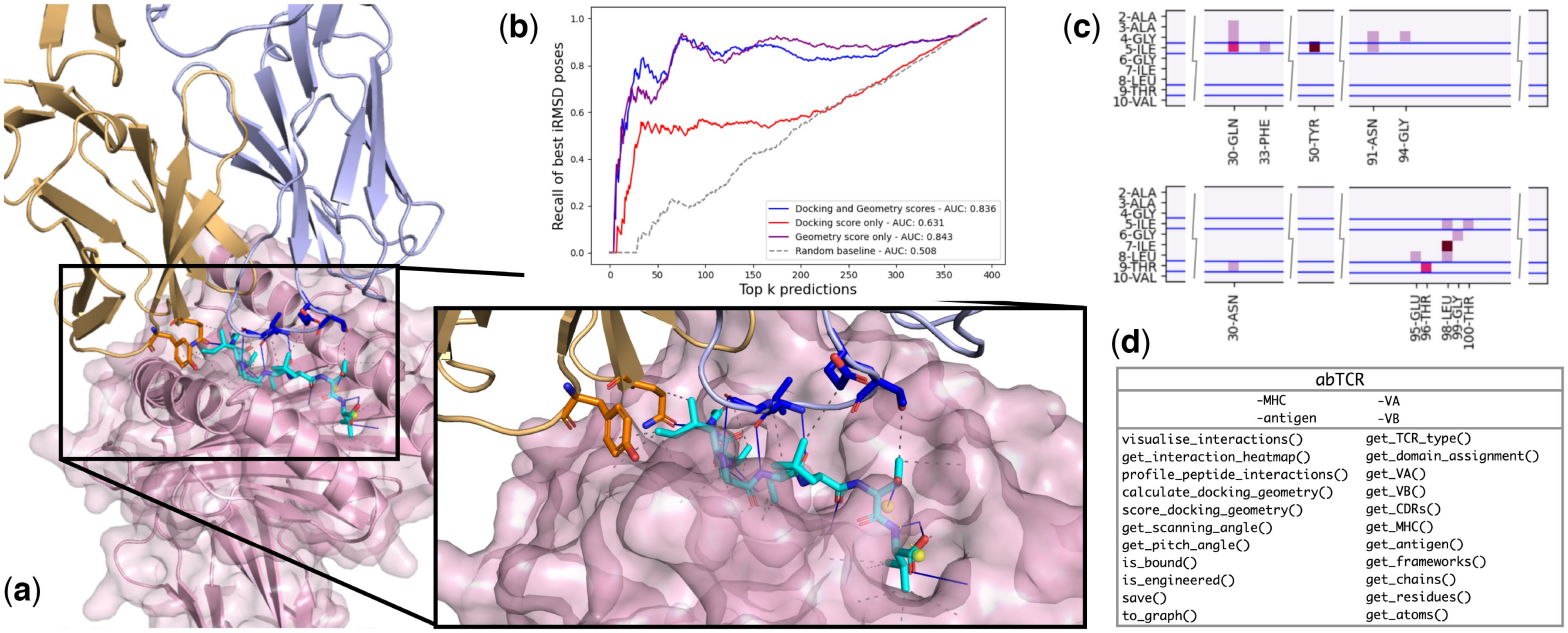
(a) Visualization of peptide interactions of αβTCR in complex with pMHC (PDB ID: 6EQB, “bulged” peptide conformation) generated with tcr.visualise_interactions() from STCRpy. Base colors: α chain in orange, β chain in lilac, MHC in pink, peptide in cyan. Interface residues are highlighted as sticks using the standard heteroatom color scheme, dashed lines depict interacting residues identified by PLIP (Adasme *et al.* (2021)). (b) Recall of lowest interface RMSD (iRMSD) TCR:pMHC complex predictions from physics-based docking using energy-based docking scores (AUC of 0.631), STCRpy geometry score (AUC of 0.843), and joint geometry and docking scores (AUC of 0.836). Random baseline as dashes (AUC of 0.508). Using STCRpy docking geometry improves recall of best predictions, combining the docking score with the geometry score improves recall at k<50. (c) An example heatmap of interactions profiled between a peptide and the TCR α chain (top), and a peptide and the TCR β chain (bottom) generated by tcr.get_interaction_heatmap() from STCRpy. Peptide residues of interest (5-ILE and 9-THR) are highlighted with bars, pixel intensity indicates the number of interactions. (d) Table of TCR object-bound attributes and methods in STCRpy.

STCRpy processes each object with PLIP (Adasme *et al.* 2021), characterizing hydrogen bonds, salt bridges, and hydrophobic or aromatic interactions within and between all protein chains. The identified interactions can be extracted as a dataframe for further analysis (e.g. pose filtering, see Applications), or visualized as a heatmap or PyMOL (Schrödinger 2015) session (Fig. 1a).

### 2.2 TCR:pMHC geometry scoring and RMSD

We implement and define the geometry of a TCR relative to the MHC using three methods, two of which have previously been reported (Rudolph *et al.* 2006, Singh *et al.* 2020), as well as an additional adaptation of the two methods, described in Appendix A, available as supplementary data at *Bioinformatics* online, thereby unifying TCR:pMHC geometry evaluations within one software package. This enables consistent, comparable and reproducible calculations of TCR to pMHC geometry. Crucially, STCRpy distinguishes poses that fit the canonical angle/polarity from those that are reverse-canonical or non-canonical modes of engagement (Beringer *et al.* 2015, Gras *et al.* 2016, Zareie *et al.* 2021), enabling systematic differentiation of poses compatible or incompatible with signaling during *in silico* analyses.

We calculated the geometry of all complexes in STCRDab whose TCRs show evidence of downstream signaling, and supply these distributions in STCRpy, providing a means of scoring or filtering candidate docks (see Section 3). Briefly, we parametrize the distribution of the scanning angle as a normal distribution, the pitch as a Gamma distribution, and the TCR to MHC distance as a mixture of Gaussians. Further, we calculate a score, η, as a linear combination of negative log-likelihood of a complex across the three distributions: $\eta =\sum_{i}-\alpha_{i}\;\text{log}(p_{i}(x_{\mathrm{TCR}:\mathrm{pMHC}}))$. We have reported the abTCR/class I distribution parameters and their estimation in Appendix A, along with the implementation of the scoring function.

STCRpy also enables fast and reproducible calculations of RMSD both between TCR structures and across interfaces of TCR:pMHC complexes against a reference structure.

### 2.3 Machine learning dataset preparation

STCRpy can parse both *apo* and bound TCR complexes into datasets of graphs using the popular pytorch-geometric and pytorch deep learning libraries (Fey and Lenssen 2019, Paszke *et al.* 2019), facilitating the use of TCR structural information in machine learning. The generated graphs are compatible with the existing pytorch-geometric suite of graph neural network architectures, and can be integrated flexibly into training paradigms of bespoke neural networks. Furthermore, STCRpy enables users to flexibly assign labels, according to the neural network architecture and task they are interested in training. Alternative node selection, node featurization, and edge featurization methods can be configured, and bespoke implementations can be passed as arguments.

## 3 Applications

### 3.1 Evaluating predicted TCR:pMHC complex poses

We first demonstrate the utility of STCRpy by scoring and retrieving accurate docks from a pool of 2000 candidates using a TCR in complex with a KRAS-G12D antigen presented by HLA-A*11:01, deposited with PDB identifier 7PB2 (Poole *et al.* 2022). As an *in-silico* complex prediction scenario, we predicted the TCR structure from sequence using TCRBuilder2+ (Quast *et al.* 2025) and Alphafold-Multimer (Evans *et al.* 2022). This resulted in nine candidate TCR structures, four from TCRBuilder2+ and five representative structures from Alphafold-Multimer, with which to initialize *in-silico* docking experiments. We report the RMSD of the TCR structure predictions, which can be easily and reproducibly calculated using STCRpy, in Appendix B Table 3, available as supplementary data at *Bioinformatics* online.

We then used a physics-based approach (HADDOCK2.4, Van Zundert *et al.* 2016) to dock the nine predicted structures and the original crystal structure of the 7PB2 TCR against the pMHC antigen. The docking simulations yielded 200 predicted TCR:pMHC complexes per TCR structure, resulting in a total of 2000 predicted TCR:pMHC complexes.

STCRpy enabled fast processing and evaluation of all predicted complexes *via* batch methods designed to handle large quantities of TCR structure data. For each pose, we used STCRpy to calculate the interface RMSD to the original crystal structure, quantify the geometry of the complexes and extract the energy-based docking scores from the HADDOCK simulations. Finally, we applied STCRpy’s geometry scoring functionality to each predicted pose.

The retrieval of plausible and accurate candidates would usually require manual inspection and evaluation, or reliance on docking scores which are relatively poor predictors of accuracy. Here, STCRpy enables consideration of both the energy-based docking score and a knowledge-based geometry score, which together correlate better with interface RMSD allowing retrieval of accurate TCR:pMHC complex predictions (Appendix B Fig. 6). Specifically, a simple linear regression of the docking and geometry score to the interface RMSD of the complex yields an AUPRC of 0.836 on the withheld validation set (Fig. 1b). Using exclusively geometry features results in a slightly higher AUC of 0.843, but worse retrieval at k<50. The docking score feature alone underperforms relative to geometric scoring (AUC of 0.631), which underpins the difficulty of using general functions for evaluating docking methods.

### 3.2 Characterizing TCR:pMHC interface interactions

Identifying the interactions between the epitope and paratope of a TCR:pMHC complex is key to understanding the binding motif and provides insights into mutations that could impact binding affinity. As a case study we characterized the interactions between the HLA-A*02:01-AAGIGILTV antigen and two TCRs, MEL5 and α24β17, deposited in the PDB with the identifiers 6EQA and 6EQB respectively (Madura *et al.* 2019). As Madura et. al. report, this antigen undergoes a shift in structural conformation from “stretched” to “bulged” upon TCR binding. We used the interaction profiling utility of STCRpy to compare the TCR:pMHC interfaces of both peptide states, showing that additional hydrophobic contacts between the TCR alpha chain and the peptide emerge in the immunogenic state (Appendix C Table 4, available as supplementary data at *Bioinformatics* online). For the full analysis, see Appendix C. The interactions, which are generated using PLIP (Adasme *et al.* 2021), can be visualized as an annotated heatmap (Fig. 1c, Appendix C Fig. 7) or in PyMOL (Schrödinger 2015, Appendix C Fig. 8).

### 3.3 Machine learning TCR property prediction

STCRpy’s TCRGraphDataset constructor converts TCR structures into graphs compatible with graph neural networks (GNNs). The graphs can be configured; by default each amino acid is defined as a node and edges correspond to distances between amino acids. We validated our graph datasets by training an equivariant graph neural network (EGNN) (Satorras *et al.* 2021) on region annotation of a point cloud, whereby the network assigns a region label, TCRα, TCRβ, peptide, or MHC, to each amino acid, based on the structure of the complex alone (see Appendix D, available as supplementary data at *Bioinformatics* online). The attained node annotation accuracies on the validation set substantially outperform the random baseline (Appendix D Table 6), demonstrating that EGNNs learn the underlying geometry of TCR:pMHC complexes from the STCRpy graph inputs.

## 4 Conclusion

We have illustrated STCRpy’s utilities (Fig. 1d) with three indicative workflows. Firstly, we have showed that STCRpy can be used to evaluate predicted TCR:pMHC complexes for TCR-likeness using a geometry score. In this case we generated poses using a physics-based approach, but STCRpy could also be applied to the outputs of machine learning models such as AlphaFold3 (Abramson *et al.* 2024). Secondly, we have demonstrated that STCRpy can be used to profile interactions between TCRs and peptides, specifically demonstrating a case where sequence analysis alone would not suffice as the interactions arise from a change in conformation. These interaction profiling methods could be exploited to generate further TCR-likeness metrics (Raybould *et al.* 2023) beyond the geometry score used in the first case study. Finally, we have shown that STCRpy can be used to convert TCR structures to graph datasets for deep learning pipelines, removing barriers to the use of predicted structural features in TCR:pMHC specificity classifiers.

Overall, STCRpy offers a simple route toward analyzing the broad diversity of structural data on TCRs and their complexes emerging from repertoire studies and TCR-based drug discovery. We have released the STCRpy codebase as an extendible open source project, and encourage community engagement and contributions as the field evolves.

## Supplementary Material

btaf566_Supplementary_Data

## Data Availability

STCRpy code is open-source and available at: https://github.com/oxpig/STCRpy. STCRDab structures used for analysis are available at: https://opig.stats.ox.ac.uk/webapps/stcrdab-stcrpred.
