# Supplementary material for: STCRpy: a software suite for T-cell receptor structure parsing, interaction profiling, and machine learning dataset preparation
Source: Bioinformatics. 2025 Oct 10;41(10):btaf566. doi: 10.1093/bioinformatics/btaf566 (PMC12574948; doi:10.1093/bioinformatics/btaf566)
Supplement: btaf566_Supplementary_Data [file btaf566_supplementary_data.pdf]

Appendix A: Geometry calculation and scoring of TCR:pMHC complexes

Geometry calculation

Three methods for calculating geometric metrics of TCR:pMHC complexes are implemented in STCRpy. All three methods calculate the scanning angle of TCR to pMHC slightly differently and we have described and characterised these differences here. The third method, which is a combination of both methods we have implemented in STCRpy, is also used to score docking geometry.

Rudolph et al.

This method for calculating the scanning angle of a TCR bound to MHC (Rudolph et al. [2006]) is implemented in STCRpy as follows:

$$\theta = acos(\frac{\vec{t} \cdot \vec{m}}{|\vec{t}||\vec{m}|}) \quad (1)$$

Where θ is the scanning angle of the TCR over the MHC, $\vec{t}$ is the vector pointing from the TCRα chain to the TCRβ and $\vec{m}$ is the vector pointing through the MHC. More specifically, the TCR vector is defined as the unit-normed vector pointing from the mean, *i.e.* centroid, of the α chain cysteine residues' sulphur atom to the mean of the β chain cysteine residues' sulphur atom, where in each chain the cysteine residues comprise the conserved variable domain disulfide bridge (between positions 23 and 104 using IMGT numbering, Lefranc et al. [2005]):

$$\vec{t} = (\frac{x_{\beta|Cys23|S} + x_{\beta|Cys104|S}}{2}) - (\frac{x_{\alpha|Cys23|S} + x_{\alpha|Cys104|S}}{2}) / |(\frac{x_{\beta|Cys23|S} + x_{\beta|Cys104|S}}{2}) - (\frac{x_{\alpha|Cys23|S} + x_{\alpha|Cys104|S}}{2})| \quad (2)$$

The MHC vector is defined as the unit-normed vector pointing through the MHC. This vector is determined as the principal component of the singular value decomposition (SVD) of the point cloud of coordinates of the MHC helices forming the peptide cleft, X.

$$\vec{m} = \vec{v}_0, X = U\Sigma V \quad (3)$$

where $\vec{v}_0$ is the first component of the V matrix of the SVD. Intuitively, v_0 explains the most variance in the coordinates X in 3D Euclidian space. The point cloud $X \in \mathbb{R}^{3\times N}$ is defined as the coordinates of the C_α of the MHC helices, translated so the centre of mass is at the origin:

$$X = \begin{bmatrix} x_1 \\ x_2 \\ \vdots \\ x_N \end{bmatrix} - \frac{1}{N}\sum_{i=0}^{N} x_i \quad (4)$$

The N residues comprising the helices are selected by residue numbering in Table 1. We use the *numpy.linalg.svd* algorithm to solve the decomposition. Finally, the sign of the vector, the direction of which is stochastic, is aligned to have the same direction as the approximate MHC vector, $\tilde{m}$, pointing from the N to the C terminus of the α_1 helix:

$$sign(\vec{m}) = sign(|\vec{m} + \tilde{m}| - |\vec{m} - \tilde{m}|) \quad (5)$$

In STCRpy, this method for calculating the scanning angle angle can be called as follows:

```
tcr.calculate_geometry(mode='rudolph').
```

Centre of mass (Singh et al. [2020])

An advantage of the Rudolph *et al.* method is that it does not require the definition of a global coordinate frame – both the TCR vector and the MHC vector are calculated within the same frame, making the angle between them invariant to rotations. However, this limits the evaluation of the TCR:pMHC geometry to metrics invariant to rotations and translations. It can also lead to spurious edge cases as the scanning angle θ is invariant to rotations of the TCR vector about the axis of the MHC vector. While this is not necessarily a limitation for well-behaved crystal structure of TCR:pMHC complexes, *in-silico* predictions often contain noisy and non-canonical predictions that this method fails to distinguish by scanning angle alone. An extreme example would be a TCR docked to an MHC from the side at a 90° angle, which would be annotated with a canonical value of θ if calculated using the Rudolph *et al.* method.

	α_1	α_2
MHC Class I	(50-87)	(140-177)
MHC Class II	(50-88)	(54-65) & (67-91)

Appendix A Table 1. MHC residue number ranges included in point cloud coordinates when calculating MHC vector $\vec{m}$ as in Eq. 3&4. Different numbering applies for MHC Class I and Class II. In Class I both helices are encoded by the same chain, whereas for Class II the alpha helices are on different chains. The two ranges for MHC Class II α_2 exclude the 'kinked' region of the second helix.

As an alternative, Singh *et al.* proposed to align TCR:pMHC complexes to MHC reference structures through which a Cartesian coordinate frame has been defined (Singh et al. [2020]). Specifically, the origin is defined to be at the centre of the peptide cleft, with the x-axis cutting across the cleft pointing from MHC helix α_1 to α_2, the y-axis running along the peptide cleft, and the z-axis pointing upwards toward the TCR interface. To calculate the TCR:pMHC complex geometry, first the TCR:pMHC complex is aligned by MHC to the correct reference structure (MHC Class I or Class II). After this alignment is complete, the TCR:pMHC sits in the predetermined global coordinate system, and absolute metrics, which are subject to translations and rotation, can be evaluated and compared across TCR:pMHC complexes.

Within this coordinate frame, we follow Singh *et al.*'s definition of the scanning angle θ, pitch ψ, and z-distance, to define the geometry of the TCR:pMHC complex. The scanning angle is defined as the angle between the projection $\tilde{t}$ of the TCR vector $\vec{t}$ onto the xy-plane and the y-axis:

$$\theta = acos(\tilde{t_y}), \begin{bmatrix} \tilde{t_x} \\ \tilde{t_y} \end{bmatrix} = \begin{bmatrix} t_x \\ t_y \end{bmatrix} / \sqrt{t_x^2 + t_y^2} \quad (6)$$

The pitch angle ψ of the TCR:pMHC complex is defined as the angle between the TCR vector $\vec{t}$ and the z-axis. Intuitively this describes the tilt of the TCR.

$$\psi = acos(\sqrt{1 - t_z^2}) \quad (7)$$

The definition of the TCR vector differs from Rudolph et.al. - instead of defining the angle pointing from the α chain to the β chain via the cysteine residues' centroid, Singh *et al.* calculate

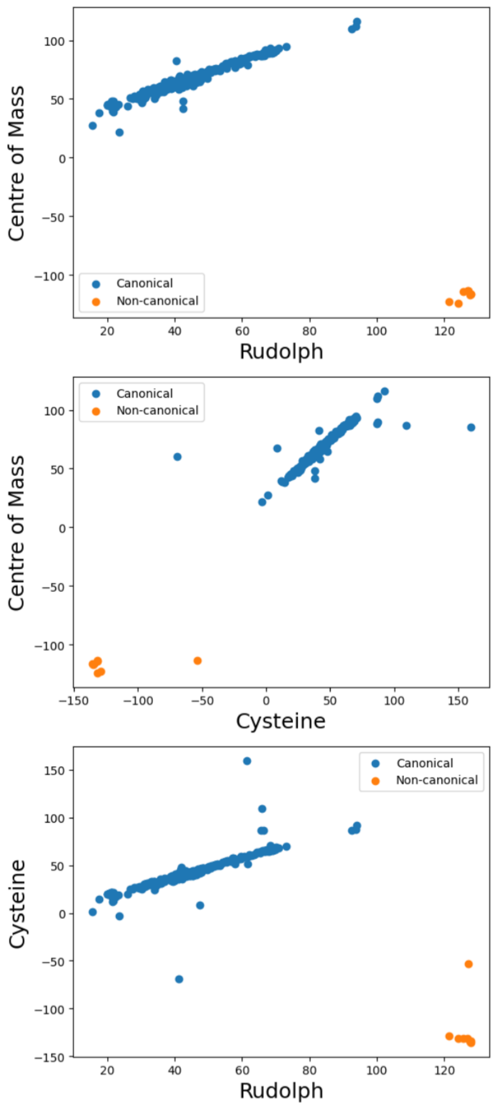

Appendix A Fig. 1. Comparison of MHC Class I TCR:pMHC scanning angles using three methods implemented in STCRpy. The methods produce linearly related scanning angles, with the exception of outliers, but the scanning angle ranges differ across methods. Non-canonical binding modes (orange) are readily separable across all three methods, with the exception of an outlier with additional engineered cysteine residues.

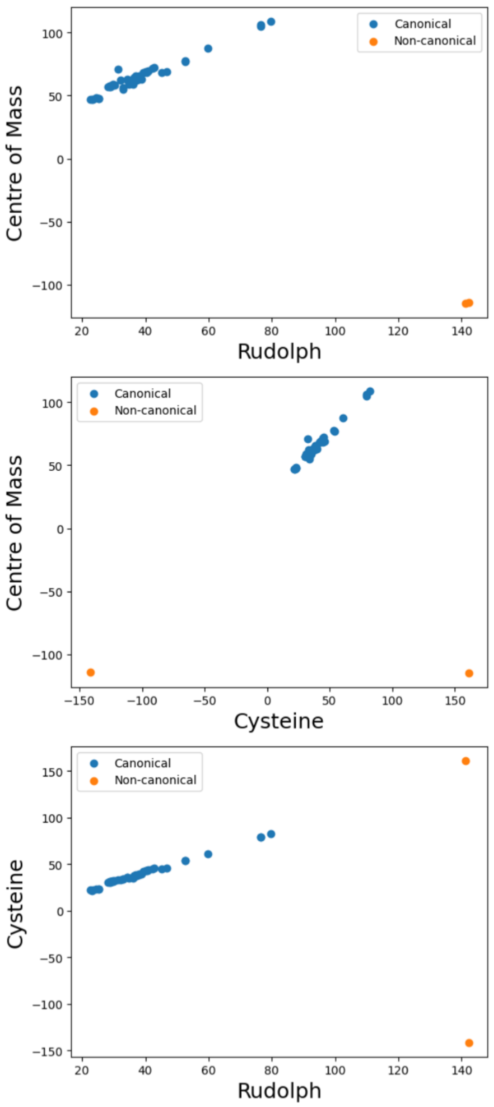

Appendix A Fig. 2. Comparison of MHC Class II TCR:pMHC scanning angles using three methods implemented in STCRpy. The methods produce linearly related scanning angles, with the exception of outliers, but the scanning angle ranges differ across methods. Non-canonical binding modes (orange) are readily separable across all three methods.

the centre of mass of a subset of each chain, and uses the vector between these points:

$$\vec{t} = \frac{CoM(\beta) - CoM(\alpha)}{|CoM(\beta) - CoM(\alpha)|} \tag{8}$$

The residue subset used for each TCR chain is provided by a reference file included in STCRpy for each TCR chain type, α, β, γ, and δ.

In STCRpy, this method for calculating the TCR:pMHC geometry can be called as follows:

```
tcr.calculate_geometry(mode='com')
```

Combined cysteine and centre of mass method

We have also implemented a new, combined way of calculating TCR:pMHC geometric features, which takes advantage of Singh *et al.*'s alignment to a global coordinate frame while avoiding the dependence on a reference residue subset for calculating the TCR centre of mass. First, we align the TCR:pMHC to a reference MHC, but then, we calculate the TCR vector using the cysteine residue centroids as in Eq. 2. The remainder of the TCR geometry is then calculated following Eqs. 6 & 7. In STCRpy, this method for calculating the TCR:pMHC geometry can be called as follows:

```
tcr.calculate_geometry(mode='cys')
```

An advantage of the global coordinate frame is the intuitive identification of canonical vs. non-canoncial TCR:pMHC binding modes: given the coordinate frame, any TCR vector with a negative x-component, ie. the vector from the TCR α chain to the TCR β chain points 'upwards' at the α_1 helix, has a canonical binding geometry. This holds true for both methods "centre of mass", and "cysteine" which align the TCR:pMHC to a global reference frame. Using the method proposed by

	Distribution type	Parameters
θ	Normal	$\mu = 67.9, \sigma^2 = 202.1$
ψ	Gamma	$\alpha = 1.20, \theta^{-1} = 5.78,$ $x = 0.0365$
$z - dist$	Gaussian Mixture	$N = 2,$ $\mu = [27.05, 28.62],$ $\sigma^2 = [0.468, 0.908],$ $w = [0.434, 0.566]$

Appendix A Table 2. Parameters of probability distributions used to calculate STCRy geometry score using maximum likelihood estimation on STCRDab data.

Rudolph *et al.* it is not possible to use absolute components of the TCR or MHC vector, since these are subject to changes under rotation and translation. However, empirically, we find that all scanning angles $\theta > 120°$ correspond to a non-canonical, reverse binding TCR:pMHC geometry. In STCRpy, the polarity can also be embedded into the scanning angle, by multiplying the angle by -1 for reverse polarity. In Fig.1&2 this has been applied to the 'cysteine' and 'centre of mass' methods.

To enable comparisons across these geometry calculations and to confirm their self-consistency, we characterised the methods using TCR:pMHC crystal structures from STCRDab (Appendix A Figs. 1 & 2).

We included all TCR:pMHC structures in STCRDab that did not raise errors due to missing residues or other rare irregularities, such as antigen joined to TCR chains, resulting in 304 MHC class I complexes from 203 unique PDB codes, and 49 MHC class II complexes from 31 unique PDB codes.

We observe that the relationship between scanning angles across all methods is linear, as expected, and that non-canonical binding modes are readily separable.

Geometry scoring

We are able to score predicted TCR:pMHC complexes by evaluating the likelihood that they arose from the known TCR:pMHC distribution. We featurise the TCR:pMHC complexes by geometric features: the scanning angle θ, the pitch angle ψ, and the z-component of the TCR centre of mass, which we calculate using the cysteine method described above. We calculate the distribution of these metrics over TCR:pMHC complexes in STCRDab, and fit simple parametric distributions to the data using maximum likelihood estimation. We then evaluate the likelihood of any query TCR:pMHC complex arising from these 'TCR-like' distributions:

$$s = w_\theta * ln(p_\theta(\hat{\theta})) + w_\psi * ln(p_\psi(\hat{\psi})) + w_z * ln(p_z(\hat{z})) \quad (9)$$

Where $\hat{\ }$ is the geometric feature of the predicted query complex. The weights w can be adjusted but are set to $w_\theta = w_\psi = w_z = 1$ by default. We approximate θ with a normal distribution, ψ with a gamma distribtuion, and z-distance with a mixture of Gaussians. We fit the distributions using the popular `scikit-learn` and `scipy` implementations. We have reported the optimal parameters in Appendix A Table 2 and visualised the fit in Appendix A Fig. 3.

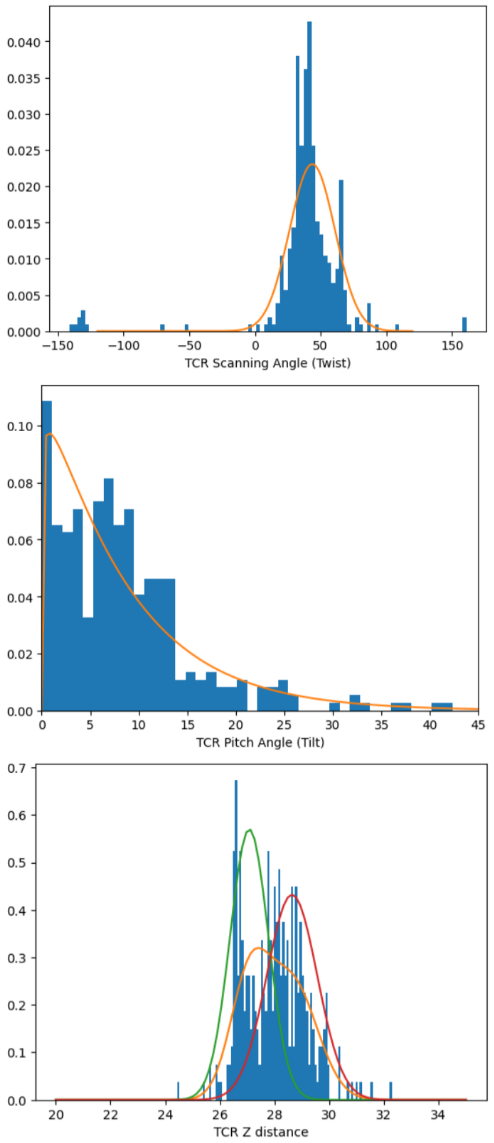

Appendix A Fig. 3. Parametric probability distributions fit to STCRDab MHC Class I data. Scanning angle θ is fit with a normal distribution, pitch angle ψ with a gamma distribution, and z-distance with a mixture of Gaussians, n=2. Blue histogram bars are STCRDab data, orange line is the maximum likelihood estimate (MLE) fit of the parametric distribution. Red and green lines are the distributions of the independent components of the Gaussian Mixture.

Appendix B: Evaluating predicted TCR:pMHC complexes

We used physics-based docking to generate 2000 TCR to KRAS-G12D HLA-A*11:01 complexes and analysed these predicted structures with STCRpy.

Results

We present the RMSD of the structure predictions used for docking in Table 3.

Plotting the scanning angle against the HADDOCK energy score demonstrates that when the TCR structure is not known, a wide range of potential TCR:pMHC complex poses are

sampled, including some which are not conducive to TCR signalling due to a non-canonical binding mode (Beringer et al. [2015], Gras et al. [2016], Zareie et al. [2021]), (Appendix B Fig. 4).

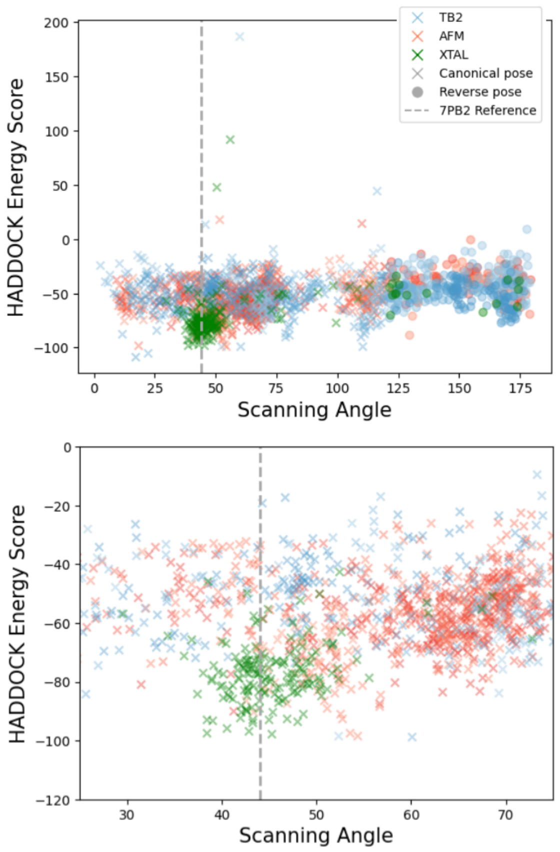

Appendix B Fig. 4. Haddock energy score of TCR:pMHC docks against scanning angle of the TCR to MHC of 7PB2 case study. Grey dashed line is the true scanning angle of the crystal structure. AFM: docks using Alphafold-Multimer TCR structure predictions, TB2: docks using TCRBuilder2+ TCR structure predictions, XTAL: docks using the experimental crystal structure of the TCR. x and o markers represent canonical and non-canonical scanning angles, respectively. When the TCR structure is predicted, rather than known, the range of predicted TCR:pMHC scanning angles increases substantially, and the docking score is approximately uniform across angles, making the retrieval of accurate or 'TCR-like' poses based on the docking score impossible.

However, when considering the interface RMSD plotted against the HADDOCK energy score and geometry scores it becomes possible to identify valid poses more readily, with lower interface RMSD clearly correlated to lower geometry scores (Appendix B Fig. 5).

To demonstrate the robustness of the geometry score as a proxy for the accuracy of a docked pose, we also evaluated the poses of four additional TCR:pMHC complexes - 6zkx, 7nme, 7rm4, 8d5q alongside the 7pb2 code we conducted the original case study on. Fig. 6 shows that the logarithm of the negative geometry score correlates with interface RMSD (PCC of 0.55 and p-value << 0.005). However, we also observe that poses with good geometry scores can still have high interface RMSD. As we show in Fig. 7, this may be related to the challenge of docking potentially inaccurate TCR structure predictions:

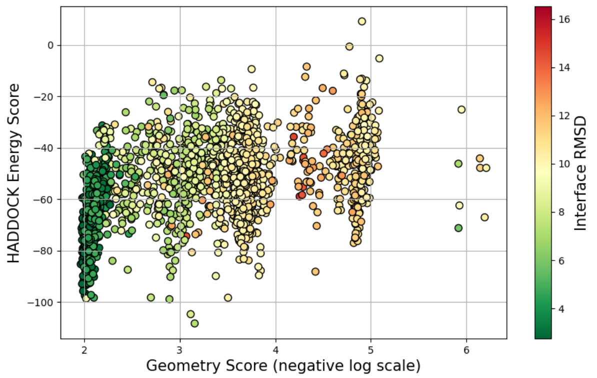

Appendix B Fig. 5. Haddock energy score *vs.* the negative natural logarithm of the STCRpy geometry score of TCR:pMHC docks, coloured by interface RMSD [Å] to the 7PB2 ground truth crystal structure. Geometry score is better correlated with low interface RMSD than the docking score.

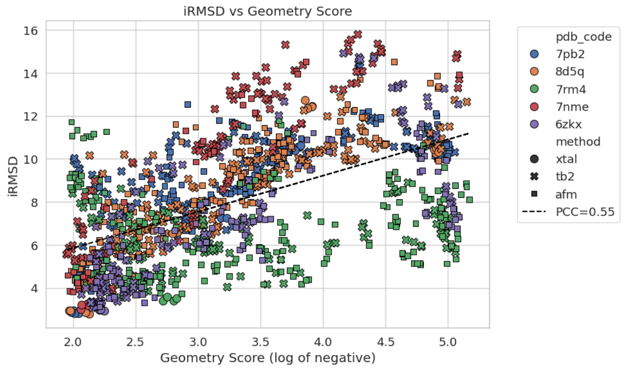

Appendix B Fig. 6. Interface RMSD (iRMSD [Å]) vs logarithm of the negative STCRpy geometry score for physics based docked poses across multiple PDB codes. TCR structures are either TCRBuilder2+ (tb2), Alphafold-Multimer (afm) predictions or crystal structures (xtal).

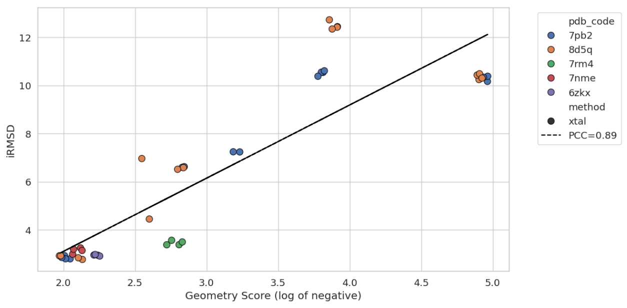

Appendix B Fig. 7. Interface RMSD (iRMSD [Å]) vs logarithm of the negative STCRpy geometry score for physics based docked poses across multiple PDB codes. Redocks of crystal structures separate display highly correlated iRMSD and geometry scores.

geometry score and interface RMSD is better correlated (PCC of 0.89 and p-value << 0.005) when considering only the redocked crystal structures.

We also used STCRpy to evaluate the complex predictions made by AlphaFold3 (Abramson et al. [2024]), which we used to predict the TCR:pMHC complex directly from sequences.

	TB2+ - 0	TB2+ - 1	TB2+ - 2	TB2+ - 3	AFM - 0	AFM - 4	AFM - 6	AFM - 12	AFM - 15
A	0.89	1.07	1.20	1.36	1.35	1.23	0.89	0.87	0.89
CDRA1	1.20	1.78	1.12	1.13	0.72	0.80	0.63	0.47	0.46
CDRA2	1.35	0.84	1.40	1.26	1.88	1.90	1.81	1.72	1.77
CDRA3	1.36	2.01	2.31	2.85	3.42	2.99	1.80	1.81	1.87
FWA	0.69	0.77	0.90	0.98	0.49	0.50	0.47	0.45	0.45
B	0.99	1.20	0.97	0.81	1.22	1.19	1.02	1.31	1.32
CDRB1	0.32	0.45	0.53	0.27	0.49	0.47	0.42	0.38	0.44
CDRB2	0.88	1.16	1.14	0.70	0.72	0.69	0.61	0.78	0.84
CDRB3	2.38	2.93	2.22	1.75	2.05	1.59	0.95	2.33	2.42
FWB	0.58	0.64	0.58	0.58	1.10	1.17	1.07	1.14	1.12

Appendix B Table 3. RMSD of TCR structure predictions used for the physics based docking case study evaluated against the 7PB2 crystal structure. TB2+: TCRBuilder2+(Quast et al. [2025]), AFM: Alphafold-Multimer (Evans et al. [2022]). '-X' denotes the internal rank of the structure prediction assigned by the prediction model. RMSD for the whole TCR and across regions can easily be calculated using: `stcrpy.tcr_metrics.RMSD().calculate_rmsd(prediction, reference)`, where `prediction` and `reference` are 'TCR' objects.

	A	CDRA1	CDRA2	CDRA3	FWA	B	CDRB1	CDRB2	CDRB3	FWB	iRMSD	Geom. score	ln(-GS)
AF3 ED rank 0	1.28	0.50	1.83	3.26	0.43	1.12	0.63	0.55	2.41	0.82	5.16	-12.65	2.53
AF3 ED rank 1	1.41	0.58	1.82	3.64	0.50	1.02	0.65	0.61	2.43	0.60	5.11	-12.24	2.50
AF3 ED rank 2	1.40	0.62	1.83	3.65	0.44	0.99	0.64	0.54	2.49	0.50	5.13	-12.86	2.55
AF3 ED rank 3	1.49	0.54	1.78	3.96	0.45	1.02	0.68	0.65	2.56	0.51	5.07	-12.19	2.50
AF3 ED rank 4	1.41	0.59	1.87	3.64	0.48	0.93	0.57	0.53	2.29	0.50	5.11	-12.47	2.52
AF3 JI rank 0	1.61	0.80	1.83	4.31	0.47	0.89	0.43	0.62	2.15	0.51	7.90	-12.02	2.48
AF3 JI rank 1	1.17	0.50	1.71	2.96	0.39	1.00	0.43	0.53	2.09	0.76	5.87	-11.10	2.40
AF3 JI rank 2	1.32	0.50	1.61	3.48	0.39	0.89	0.25	0.44	1.51	0.80	8.37	-10.44	2.34
AF3 JI rank 3	1.53	0.81	1.95	4.04	0.40	0.85	0.30	0.44	1.63	0.70	8.44	-10.93	2.39
AF3 JI rank 4	1.48	0.61	1.97	3.85	0.47	0.92	0.20	0.48	1.48	0.85	5.67	-14.28	2.65
Mean	1.41	0.60	1.82	3.68	0.44	0.96	0.48	0.54	2.10	0.65	6.19	-12.12	2.49

Appendix B Table 4. Evaluation metrics of complex TCR structure predictions made by AlphaFold3. RMSD by TCR region, iRMSD, geometry score, and the logarithm or the negative geometry score (ln(-GS)).

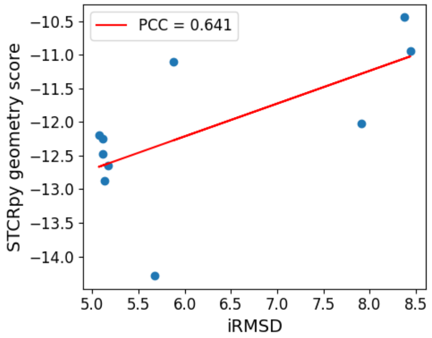

Appendix B Fig. 8. STCRpy geometry score against interface RMSD of AlphaFold3 predictions of the 7PB2 TCR:pMHC complex. Linear regression fit with correlation coefficient of 0.641 and p of 0.0459 < 0.05.

We report the RMSD of the TCR regions, the interface RMSD, and the docking geometry scores in Table 4. The relationship between the STCRpy generated geometry score and the interface RMSD (Fig. 8) of the AlphaFold3 predictions shows strong positive correlation (PCC of 0.641 with p of 0.0459 < 0.05), implying that geometry scoring remains a useful tool for filtering proposed TCR:pMHC complex poses even when the modelling tool has a data-driven prior for TCR-like complex poses.

Methods

We predicted the TCR structure of the 7PB2 TCR using TCRBuilder2+ (Quast et al. [2025]) and Alphafold-Multimer (Evans et al. [2022]), retrieving four and 25 predictions from each model respectively. For the Alphafold-Multimer structures we clustered the structures into distinct conformations with greedy clustering and a 1Å threshold, yielding 5 distinct predictions. We used these five predictions in addition to the four TCRBuilder2+ predictions for docking.

We prepared the structures for docking by separating the antigen from the TCR of the 7PB2 crystal structure. We then used the reformatting functions implemented in SCTRpy to renumber the structures and assign all residues to single chains, making them compatible with the docking software, HADDOCK2.4 (Van Zundert et al. [2016]). We defined the CDR loops of the TCR and the peptide residues as 'active' residues in the simulation; HLA residues within 6Å of the peptide were defined as 'passive' residues. The remaining parameters of the HADDOCK simulations were left as default. We docked the nine structure predictions and the TCR crystal structure to the antigen structure, yielding 2000 predicted TCR:pMHC complexes in total.

After the docking simulations were complete we used the STCRpy reformatting methods to restore the numbering of the TCR:pMHC complexes and parsed docking metrics, such as the energy-based docking score from the simulation. We then calculated the interface RMSD to the crystal structure of the complex, the scanning angle, pitch angle, and z-distance (using the 'centre-of-mass' method) of the TCR relative to the antigen, as well as the geometry scores of each predicted

complex. We further profiled the peptide interactions of every complex. Using STCRpy this analysis reduces to a single line, which saves the retrieved metrics to CSV files at a specified location:

```
stcrpy.tcr_methods.tcr_batch_operations.
    analyse_tcrs(
            tcr_complex_files,
            save_dir=".")
```

To retrieve a docking complex from the 2000 candidates, we rejected any TCR:pMHC complexes where no interactions were profiled between the peptide and the TCR. We then rank the remaining docks by their geometry score and select the candidate with the lowest score.

To investigate the retrieval power of the docking scores and geometry scores, we used a simple linear regression model to predict the interface RMSD, $iRMSD$. The linear regression was fit on 80% of the predicted TCR:pMHC complexes. We fit three models, one with both the energy based docking term, x_D, and the natural logarithm of the negative geometry score, $ln(-x_g)$. We then ranked the predicted interface RMSD of the remaining 20% of complexes, and calculated the recall of the top k predictions as $recall(k) = |Top_k(iRMSD) \cap Top_k(i\widehat{RMSD})|/k$, where $i\widehat{RMSD}$ is the predicted interface RMSD. We report the AUC of the recall curve calculated as $AUC = \sum_{k=1}^{V} recall(k)$, where V is the size of the validation set.

To generate the AlphaFold3 predictions we used the STCRpy formatting tool:

```
stcrpy.tcr_formats.tcr_formats.to_AF3_json(
    tcr, V_domain_only=True)
```

to convert TCR objects to json files compatible with the AlphaFold3 server. We used version 1 of AlphaFold3 with no structure templates to prevent the model from retrieving the original crystal structure as a template.

Appendix C: Interaction profiling of 6EQA and 6EQB

Results

Using STCRpy, we have analysed the ‘bulged’ and ‘stretched’ conformations of the 6EQA and 6EQB TCR:pMHC complexes’ interaction profiles. We show the interaction heatmaps in Appendix Fig. 9, as well as the visualised interactions of the structures in Appendix Fig. 10. We have quantified the interactions profiles in Appendix Table 5. As reported in the original paper (Madura et al. [2019]), the TCR-induced ‘bulged’ state of the peptide results in additional interactions between the TCR and antigen, which are expected to facilitate binding and signalling of the TCR.

	MEL5		$\alpha 24\beta 17$	
	Bulged	Stretched	Bulged	Stretched
hbond	4	0	5	0
hydrophobic	1	1	2	1
saltbridge	0	0	0	0

Appendix C Table 5. Interactions between the TCR (MEL5 and $\alpha 24\beta 17$) and the peptide in both stretched and bulged conformations as profiled by *STCRpy* using PLIP (Adasme et al. [2021]). More interactions between the peptide and TCR emerge in the bulged peptide conformation.

Methods

TCR:pMHC crystal structures deposited in the PDB with the identifiers 6EQA (MEL5) and 6EQB ($\alpha 24\beta 17$) with the stretched and bulged conformations recorded as alternative coordinates with the PDB record. After separating the conformations, we use the following STCRpy functions to profile and visualise the interactions:

```
# load TCR structure and profile interactions
tcr_complex = stcrpy.load_TCR(PDB_FILE)
interactions_dataframe =
    tcr_complex.profile_peptide_interactions()
tcr_complex.get_interaction_heatmap()
tcr_complex.visualise_interactions()
```

To demonstrate the flexibility of STCRpy we adjust the thresholds of the interaction identifier; the full interface can be found in the package documentation. In this study we use the parameter configurations specified in Appendix C Table 6.

	d_{BS}^{max}	d_{vdw}^{max}	d_{Hbond}^{max}
Nr. detected interactions (Tab. 5)	7.5	4.0	4.1
Interaction heatmap (Fig. 9)	10.	7.5	6.0
3D visualisation (Fig. 10)	10.	5.	4.5

Appendix C Table 6. Parameter configurations used for profiling interactions. All units in Ångström. d_{BS} - maximum distance from the peptide at which to include binding site residues; d_{vdw}^{max} - maximum distance at which to detect hydrophobic contacts; d_{Hbond}^{max} - maximum distance between hydrogen bond donor and acceptor. All other interaction parameters were set to their default values, for more details see the *STCRpy* documentation.

Appendix D: Graph Machine Learning with STCRpy

STCRpy can generate graph objects and datasets compatible with graph neural networks in deep learning pipelines. We validated our graph datasets by training the popular equivariant graph neural network (EGNN) architecture on a region annotation task, whereby the model classifies amino acids in the graph as belonging to the TCRα, TCRβ, peptide, or MHC chains.

Results

Region	EGNN	Random Baseline
TCR α	94.68%	37.43%
TCR β	96.30%	47.87%
Peptide	84.30%	12.73%
MHC	82.56%	2.08%

Appendix D Table 7. Node classification accuracy on validation set for each TCR:pMHC region.

We report the node classification accuracies for region annotation in Appendix D Table 7. The EGNN attains good accuracies considering the size of the network, particularly on peptide and MHC residue annotation, which are relatively undersampled in the dataset. As the EGNN retains equivariance by considering the relative distances between

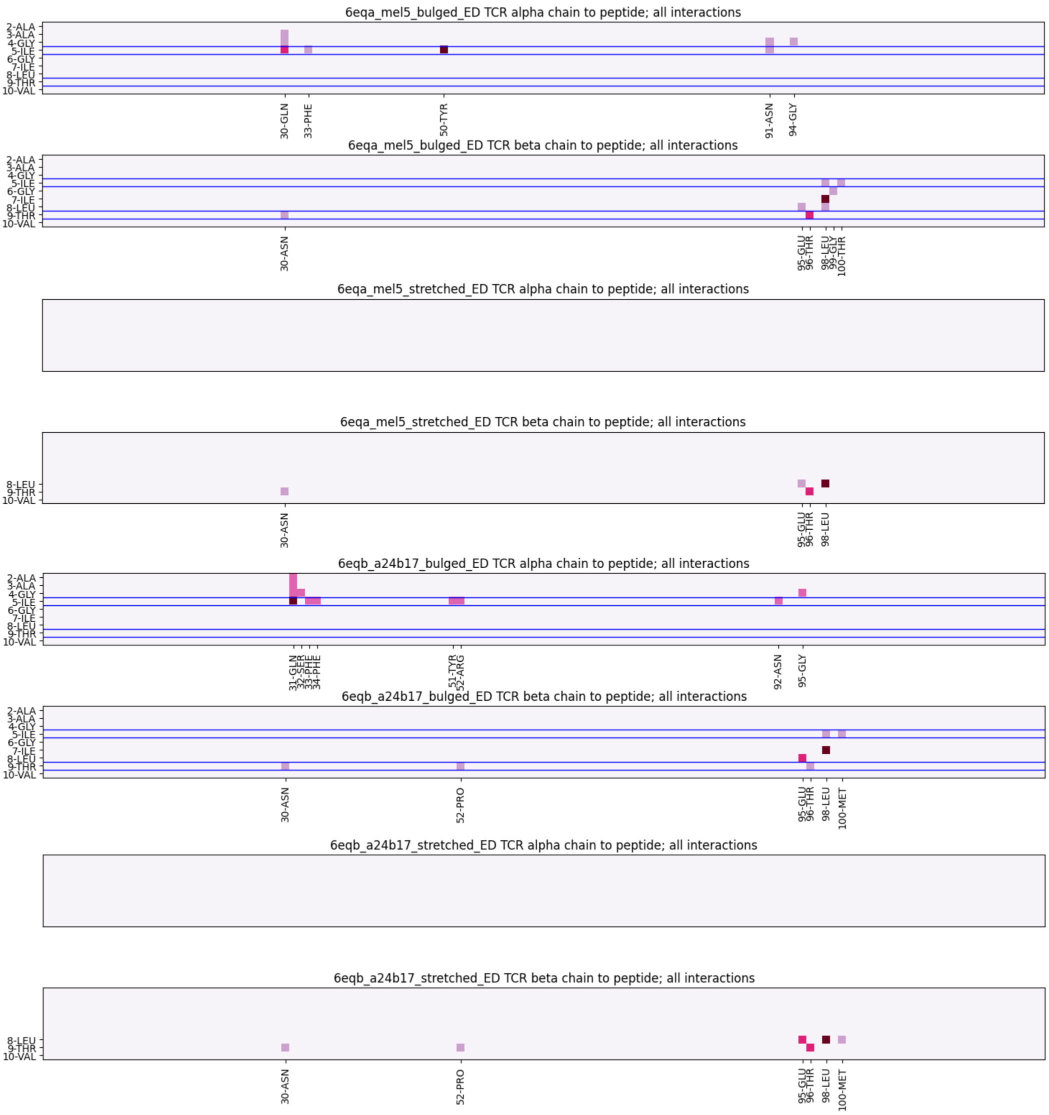

Appendix C Fig. 9. STCRpy generated TCR to peptide interaction heatmaps of 6EQA and 6EQB complexes in both the 'bulged' and 'stretched' peptide conformations. Pixel intensity indicates the number of profiled interactions. As reported by Madura et al. [2019], additional interactions emerge between the peptide and the TCR in the bulged peptide conformation. Specifically, interaction between the CDRα chains and the proximal peptide residues 2-ALA through to 5-ILE emerge.

coordinates to update node features, the ability of a small network to recapture the regions implies that the graph representation of TCR:pMHC complexes encodes structural information specific to these complexes. For context, the random baseline is achieved by sampling node classes randomly from a biased distribution, where the bias is determined by the relative frequencies of node labels.

Methods

We use the STCRpy graph dataset generator to re-format TCR structures from STCRDab (Leem et al. [2018]) to `torch_geometric` compatible graphs. Node and edge featurisation is flexible, here we embed the residue and atom type as a one-hot encoding, and pass the atom coordinates as node associated positions. We opt to define a fully-connected graph with binary edges, since the EGNN calculates the distance and selects a maximum number of nodes with which to update the nearest neighbours internally. To reduce the graph size we opt to include only the carbon alpha of each residue, and only include MHC residues within 15 Ångström of any TCR residues. The final graph representation is akin to a point cloud of amino acids coordinates, with edges corresponding to their pairwise distances. We labelled each node by region (TCR α, TCR β, peptide, or MHC) and used these annotations as the classification target. We use a random 3:1 split for training and validation, resulting in 319 and 107 training and validation structures, respectively.

We built an equivariant graph neural network using the `egnn` package (Satorras et al. [2021]). Specifically, we define a linear node embedding layer, which projects the one-hot node encodings into 32 dimensions. We then pass the embeddings and the residue coordinates through three equivariant graph network layers, along with the fully-connected adjacency matrix. The graph layers constitute a message passing

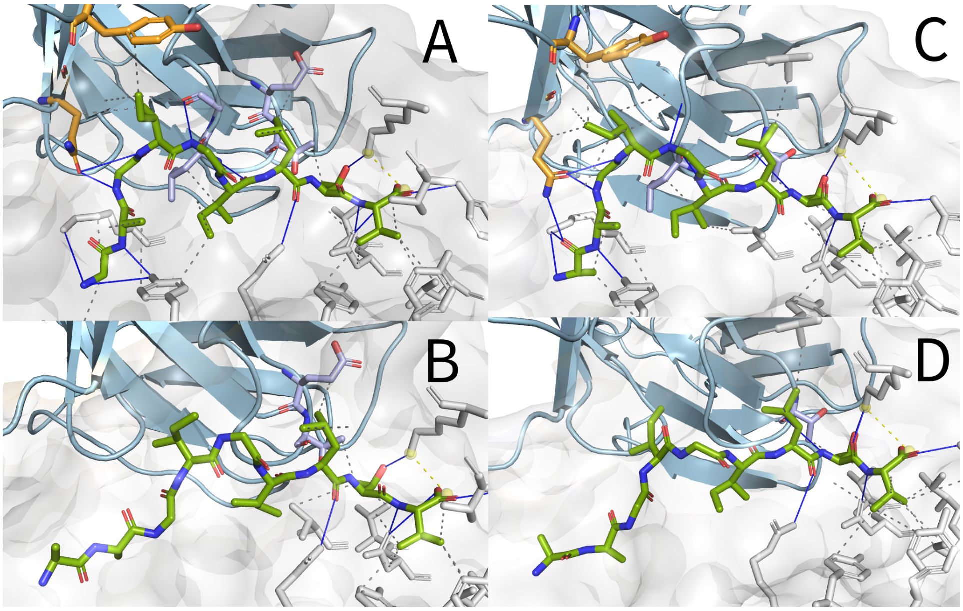

Appendix C Fig. 10. Peptide interactions of 'bulged' (A & C) and 'stretched' (B & D) peptide conformations visualised using STCRpy interface for PLIP (Adasme et al. [2021]) and PyMOL (LLC and DeLano [2020]). Peptide in green, MHC in grey, TCR in blue. Interacting TCR α and β chain residues in orange and lilac respectively.

network that achieves SE(3) equivariance by considering only the relative distance between nodes rather than explicit coordinates, for details, refer to Satorras et al. [2021]. We select a maximum of 16 neighbouring nodes to be included in each node update, and use an internal node embedding dimension of 32. As we are performing node classification we do not update the coordinate positions as the graph proceeds through the network. We pass the final node embeddings through an output network consisting of two linear layers with an intermediate ReLU nonlinearity to obtain the class probabilities as logits. We trained the network using weighted cross-entropy loss and the Adam optimiser without weight decay. We defined the cross-entropy weights as the inverted frequency of classes in a random sample: $w = [2.6471, 2.0886, 7.9839, 55.0000]$, thereby increasing the weight of the under-represented classes. We trained the network to convergence on the validation set.

References

J. Abramson et al. Accurate structure prediction of biomolecular interactions with AlphaFold 3. *Nature*, 630: 493–500, 6 2024. doi: 10.1038/s41586-024-07487-w.

M. F. Adasme et al. PLIP 2021: Expanding the scope of the protein-ligand interaction profiler to DNA and RNA. *Nucleic Acids Research*, 49:530–534, 7 2021. doi: 10.1093/nar/gkab294.

D. X. Beringer et al. T cell receptor reversed polarity recognition of a self-antigen major histocompatibility complex. *Nature Immunology*, 16, 2015. doi: 10.1038/ni.3271.

R. Evans et al. Protein complex prediction with AlphaFold-Multimer. *bioRxiv*, 3 2022. doi: 10.1101/2021.10.04.463034.

S. Gras et al. Reversed T Cell Receptor Docking on a Major Histocompatibility Class I Complex Limits Involvement in the Immune Response. *Immunity*, 45, 2016. doi: 10.1016/j.immuni.2016.09.007.

J. Leem et al. STCRDab: the structural T-cell receptor database. *Nucleic acids research*, 46:406–412, 1 2018. doi: 10.1093/NAR/GKX971.

M. P. Lefranc et al. IMGT, the international ImMunoGeneTics information system®. *Nucleic Acids Research*, 33, 1 2005. doi: 10.1093/nar/gki065.

S. LLC and W. DeLano. The PyMOL Molecular Graphics System, Version 2.0 Schrödinger, LLC., 2020.

F. Madura et al. TCR-induced alteration of primary MHC peptide anchor residue. *European Journal of Immunology*, 49, 2019. doi: 10.1002/eji.201948085.

N. P. Quast et al. T-cell receptor structures and predictive models reveal comparable alpha and beta chain structural diversity despite differing genetic complexity. *Communications Biology*, 8:362, 3 2025. doi: 10.1038/s42003-025-07708-6.

M. G. Rudolph, R. L. Stanfield, and I. A. Wilson. How TCRs bind MHCs, peptides, and coreceptors. *Annual Review of Immunology*, 24, 2006. doi: 10.1146/annurev.immunol.23.021704.115658.

V. G. Satorras, E. Hoogeboom, and M. Welling. E(n) Equivariant Graph Neural Networks. In *Proceedings of Machine Learning Research*, volume 139, 2021.

N. K. Singh et al. Geometrical characterization of T cell receptor binding modes reveals class-specific binding to maximize access to antigen. *Proteins: Structure, Function and Bioinformatics*, 88, 2020. doi: 10.1002/prot.25829.

G. C. Van Zundert et al. The HADDOCK2.2 Web Server: User-Friendly Integrative Modeling of Biomolecular Complexes. *Journal of Molecular Biology*, 428:720–725, 2 2016. doi: 10.1016/j.jmb.2015.09.014.

P. Zareie et al. Canonical T cell receptor docking on peptide-MHC is essential for T cell signaling. *Science*, 372, 2021. doi: 10.1126/science.abe9124.
